# Learning with the Spinal Cord

**DOI:** 10.1371/journal.pbio.1002187

**Published:** 2015-06-30

**Authors:** Richard Robinson

**Affiliations:** Freelance Science Writer, Sherborn, Massachusetts, United States of America

## Abstract

To what extent does the spinal cord play a role in the learning of motor tasks? A new study that simultaneously images the brain and spinal cord shows that the spinal cord is actively and independently involved in the earliest stages of motor learning.

Learning a complex motor task, such as mastering the fingering of a Bach partita, produces significant changes in synaptic connections in the brain’s motor control areas, including the sensorimotor strip along the top of the brain’s cortex, and the cerebellum, the small cauliflower-shaped subdivision at the rear. While the spinal cord receives motor commands and transmits them out to the muscles, whether or not it plays an independent role in motor learning has been an open question, in part because of an absence of tools to distinguish plastic changes intrinsic to the cord itself from changes dictated by alterations in the brain.

Those difficulties have been overcome in a new study from Shahabeddin Vahdat, Ovidui Lungu, Julien Doyon, and colleagues, who use simultaneous functional magnetic resonance imaging (fMRI) of the brain and spinal cord to show that motor output areas of the cord that control the finger muscles display learning-related changes in blood flow independent of those in the brain while learning a complex finger movement.

Subjects in the study lay in an advanced MRI machine with a field of view long enough to capture images from the brain and, at the same time, from the cervical spinal cord, where signals to and from the hand muscles come and go. Subjects performed two finger-tapping tasks, one simple (tapping the fourth, third, second, and first fingers in succession) and one complex (tapping the fourth, first, third, second and fourth fingers). The speed with which they improved on each task was recorded, as well as the changes in blood oxygen level-dependent (BOLD) signal from various regions in the brain and spinal cord as they learned the task. Captured by the MRI detector, BOLD signals reflect the moment-by-moment demand for oxygen by active neurons (Fig 1).

**Fig 1:**
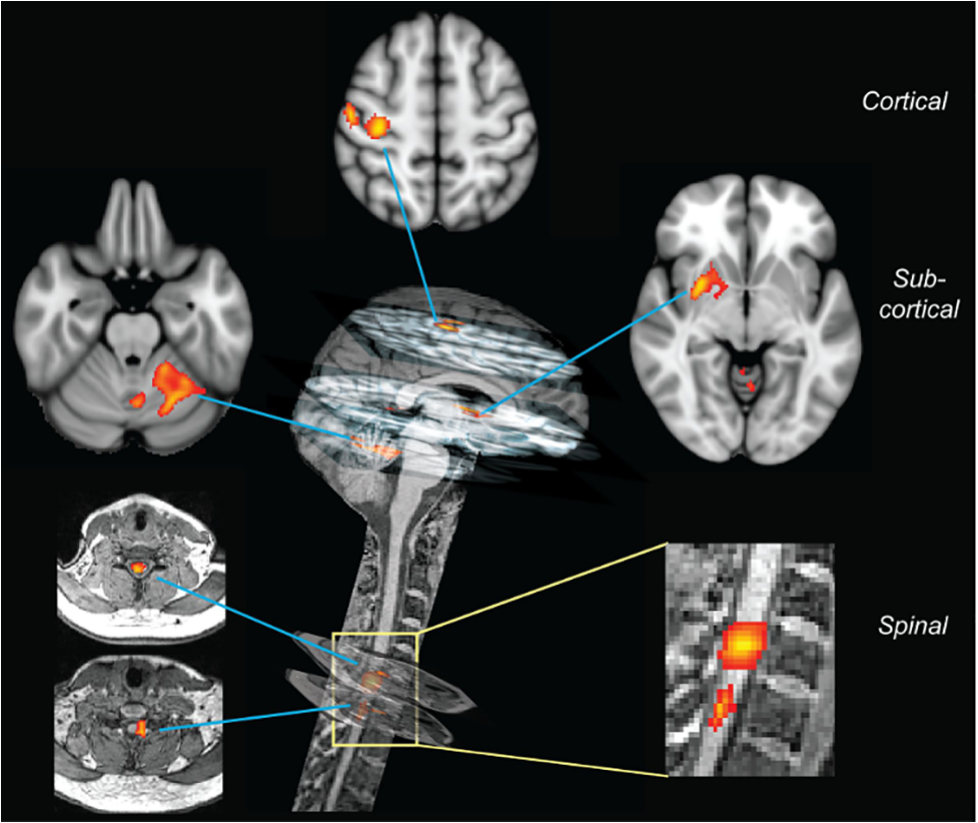
Learning-dependent plasticity at all levels of the central nervous system. Distinct cortical, subcortical, and spinal cord regions show changes in BOLD activity (color-coded in red/yellow) associated with learning of a complex finger sequence. *Image credit*: *Shahab Vahdat*.

Differences in the learning curves between the two tasks suggested that improvements in the simple task reflected practice alone, while improvements in the complex task also reflected learning the unfamiliar sequence. That larger effort was accompanied by a larger BOLD signal from the cervical cord during performance of the complex task and by greater changes in the BOLD signal over the course of learning.

Of course, the brain was also active during the task, and BOLD signals from multiple regions also changed during the learning process. The authors performed a variety of statistical analyses on images from the brain and spinal cord to determine how one correlated with the other. They found that learning-related signal changes in the spinal cord were independent of signals from either those cortical areas that project neurons to the spinal cord or those areas that showed learning-related BOLD signal changes themselves, implying that the changes in the cord were not under strict control from the brain but rather were intrinsic to the cord itself. They also found that the involved cervical regions became more influenced by the cerebellum and less influenced by the cortex as learning proceeded, in keeping with the known role of the cerebellum as one of the main coordinators of learned motor programs.

The results here indicate that the spinal cord plays an active role in the very earliest stages of motor learning. While future studies will be needed to determine whether the plastic changes seen here persist and thus become part of a distributed network of motor memory, there seems little reason to doubt it. The finding of significant independent plasticity in the cord may have important implications for rehabilitation strategies after spinal cord injury, opening up the possibility of tapping that learning ability directly to improve post-injury motor function.
